# Case Report: Dual Target Deep Brain Stimulation With Externalized Programming for Post-traumatic Complex Movement Disorder

**DOI:** 10.3389/fnins.2021.774073

**Published:** 2021-11-08

**Authors:** Ron Gadot, Ben Shofty, Ricardo A. Najera, Adrish Anand, Garrett Banks, Abdul Basit Khan, Melissa A. LoPresti, Nora Vanegas Arroyave, Sameer A. Sheth

**Affiliations:** ^1^Department of Neurosurgery, Baylor College of Medicine, Houston, TX, United States; ^2^Department of Neurological Surgery, Columbia University Medical Center, New York, NY, United States; ^3^Department of Neurology, Baylor College of Medicine, Houston, TX, United States

**Keywords:** deep brain stimulation, trauma, tremor, movement disorder, DRTT = dentato-rubro-thalamic tract

## Abstract

**Introduction:** Movement disorders can be common, persistent, and debilitating sequelae of severe traumatic brain injury. Post-traumatic movement disorders are usually complex in nature, involving multiple phenomenological manifestations, and can be difficult to control with medical management alone. Deep brain stimulation (DBS) has been used to treat these challenging cases, but distorted brain anatomy secondary to trauma can complicate effective targeting. In such cases, use of diffusion tractography imaging and inpatient testing with externalized DBS leads can be beneficial in optimizing outcomes.

**Case Description:** We present the case of a 42-year-old man with severe, disabling post-traumatic tremor who underwent bilateral, dual target DBS to the globus pallidus internus (GPi) and a combined ventral intermediate nucleus of the thalamus (Vim)/dentato-rubro-thalamic tracts (DRTT) target. DRTT fiber tracts were reconstructed preoperatively to assist in surgical targeting given the patient’s distorted anatomy. Externalization and survey of the four leads extra-operatively with inpatient testing allowed for internalization of the leads that demonstrated benefit. Six months after surgery, the patient’s tremor and dystonic burden had decreased by 67% in the performance sub-score of The Essential Tremor Rating Scale (TETRAS).

**Conclusion:** A patient-tailored approach including target selection guided by individualized anatomy and tractography as well as extra-operative externalized lead interrogation was shown to be effective in optimizing clinical outcome in a patient with refractory post-traumatic tremor.

## Introduction

Movement disorders may be a chronic, disabling sequelae of severe traumatic brain injury (TBI) (Krauss and Jankovic, 2002). Following severe TBI, 13–66% of patients have persistent and disabling movement disorders including various combinations of tremors and dystonia (Krauss and Jankovic, 2002). Stereotactic surgery is an effective form of therapy for these difficult to treat disorders with thalamotomy and deep brain stimulation (DBS) demonstrating variable but often long-term functional benefits (Krauss and Jankovic, 2002; Rojas-Medina et al., 2016; Artusi et al., 2018; Mendonça et al., 2018). Given the variable movement disorder phenomenology in TBI, it is likely that the underlying pathophysiology involves multiple motor circuits. Thus, in addition to the distorted anatomy in these patients, it is often unclear which stimulation targets are ideal. The ventral intermediate nucleus of thalamus (Vim), globus pallidus internus (GPi), and ventral oralis posterior/anterior of thalamus (VOP/VOA) are some of the more widely used targets (Espinoza Martinez et al., 2015; Rojas-Medina et al., 2016; Artusi et al., 2018; Mendonça et al., 2018). Distortion of standard anatomy portends a complexity which necessitates patient-specific lead targeting and implantation protocols that maximize symptom improvement while minimizing adverse outcomes. Here, we present the case of a patient with severe post-traumatic posture and action tremor who was implanted with 4 DBS electrodes (bilateral GPi, bilateral Vim) with 2 of 4 contacts in the bilateral Vim leads advanced ventrally to partially cover the dentato-rubro-thalamic tract (DRTT) fibers. We describe the use of personalized diffusion tensor imaging (DTI) tractography in robot-based stereotactical planning as well as inpatient, externalized lead testing to determine optimal target selection and stimulation parameters. Treatment outcome and a discussion of the literature is presented.

## Case Description

The patient has given informed consent for the publishing of all materials presented. This report was done under CARE (Consensus-based Clinical Case Reporting) guidelines to ensure completeness and transparency.

### Pre-operative Assessment

A 42-year-old right-handed man with a history of diffuse axonal injury secondary to a motor vehicle accident 24 years prior presented for evaluation and treatment of severe, disabling post-traumatic tremor. At the time of injury, he spent 5 weeks intubated in a coma and experienced retrograde amnesia for an additional period of 6 weeks. A CT scan from the time of the accident showed bifrontal contusions and evidence of sheer injury in the bilateral cerebral hemispheres, corpus callosum, and cerebellum. Several months following the accident, he began developing intermittent posture and action tremor of his hands and head. He was later diagnosed with cerebellar outflow tremor.

He presented to our clinic for consideration of DBS surgery. At presentation, he reported persistent tremor affecting both hands, left more than right, impairing his ability to complete daily manual activities including eating with utensils, drinking from a cup, and using digital devices. While there was no consistent resting tremor, he reported prominent tremor with posture and action. He endorsed bilateral leg tremors that did not affect his ability to walk but resulted in poor balance and frequent falls. He experienced titubation and head tremor that had partially improved over the years. Associated issues included severe dysarthria, irritability, anxiety, and depression. He experienced partial tremor benefits from primidone but had failed other pharmacologic therapies including propranolol, clonazepam, topiramate, oxcarbazepine, and baclofen as well as botulinum toxin injections.

**FIGURE 1:**
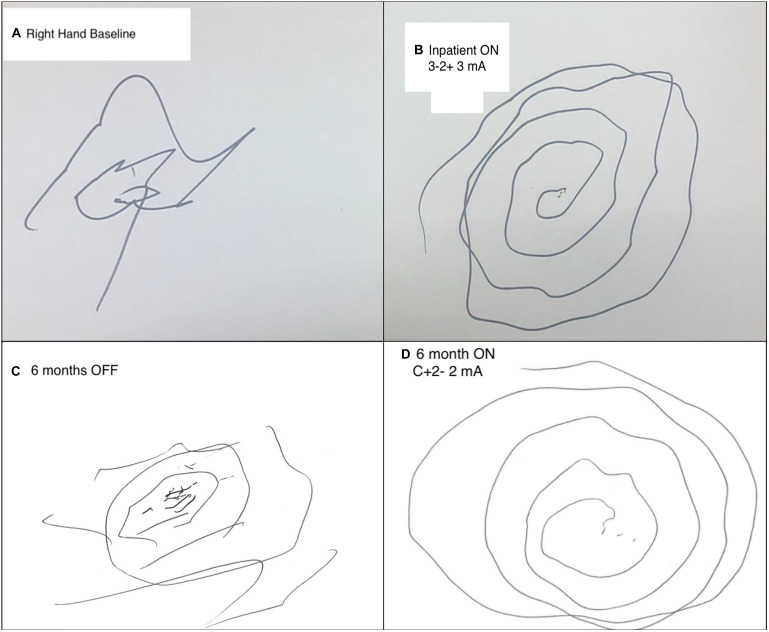
Comparison of pre- and post-operative spiral draw testing. **(A)** Preoperative baseline attempt at Archimedes spiral drawing with the right hand severely limited by tremor burden. **(B)** Spiral drawing with the right hand during inpatient extra-operative externalized testing. Left Vim configured on bipolar (3-2+) settings at 3 mA, 60 μs, and 130 Hz. **(C)** 6-month post-operative spiral drawing with the right hand OFF Vim stimulation. **(D)** 6-month post-operative spiral drawing with L Vim turned on final setting configuration: 2 mA, 70 μs, and 150 Hz.

His examination was significant for postural and action tremor of both hands, greater on the left. Arm tremor was characterized by high (and variable) amplitude, low frequency, and predominant involvement of proximal segments of his upper extremities. In addition, there was a prominent wing-beating component, and exacerbation of the tremor during hand flexion. There was moderate dysmetria on finger-to-nose testing both proximally and distally. As depicted in Figure 1A, tremor markedly affected spiral drawing. There was mild head tremor and truncal titubation at rest. His gait was dystonic with increased stride length on the left side. MRI and CT at presentation revealed marked cerebral volume loss, expansion of the ventricular and extra-axial CSF spaces, and left greater than right anterior frontal encephalomalacia.

His case was discussed at our multidisciplinary DBS case conference, including neurosurgeons, neurologists, and neuropsychologists. Given his lack of improvement with pharmacotherapy, the team agreed that surgical intervention may provide a benefit. Special consideration was given to the observed post-traumatic anatomical changes, including alteration of basal ganglia anatomy, ex-vacuo enlargement of the ventricular system further leading to anatomical distortion, and atrophy of several structures including the thalamus, GPi and the cerebral peduncles. These anatomical changes influenced stereotactic planning in target selection. The nature of his tremor and presence of dystonia were suggestive of both cerebellar and basal ganglia dysfunction. Due to his anatomical and symptomatic complexity, there was question about optimal targeting for maximal symptom improvement. His dominant postural and kinetic tremor suggested indication for bilateral Vim targeting. However, due to thalamic anatomical distortion on preoperative imaging as well as dystonic and bradykinetic components of his disorder, GPi targeting was hypothesized to confer potential benefit. Thus, an approach was planned to implant two stimulation targets concurrently to reduce the chance for treatment failure and obviate the need for repeat surgery at a later date. Bilateral Vim and GPi were targeted with planned lead externalization for inpatient testing. This would potentially aid in the optimization of target selection before stage 2 internalization and connection of effective leads to implanted pulse generators (IPGs). This strategy would allow for exploration of the effects of each of the 4 leads with possible reposition or removal of leads found to be ineffective in alleviating symptoms.

### Operation

**FIGURE 2:**
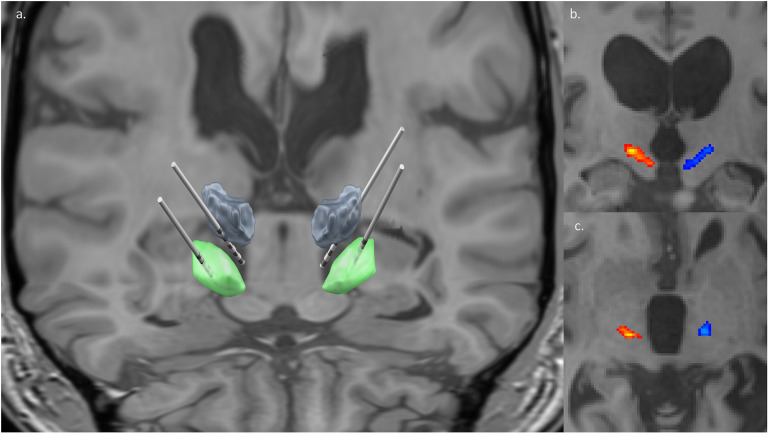
Lead and white matter tract reconstruction. **(a)** Four DBS [bilateral Vim/DRTT (gray), bilateral GPi (green)] leads reconstructed with patient’s preoperative T1 non-contrast MRI as backdrop and DISTAL atlas segmentations of deep brain nuclei. Two distal contacts of Vim leads can be seen intentionally deep to the nucleus, targeting white matter fiber tracts. Lead visualization generated in Lead-DBS (Horn et al., 2019). **(b,c)** The right (red) and left (blue) DRTT (dentato-rubro-thalamic tract) are overlaid onto the patient’s T1 non-contrast MRI. The coronal slice **(b)** showcases the tracking course of the DRTT, while the axial slice **(c)** captures the DRTT along the AC/PC plane. Tractography images generated in FSL.

The patient was brought to the operating room and induced under general anesthesia, as cooperation with awake testing was thought to be challenging in his case. A Leksell (Elekta, Atlanta, GA, United States) stereotactic head frame was placed, and a fluoroscopic CT was performed (O-arm O2 Medtronic, Minnesota, United States). The images were volumetrically fused with preoperative planning CT and MRI using ROSA (Zimmer Biomet) robotic navigation software. For further details regarding our robotic stereotactic workflow, see Karas et al. (2020). Using preoperatively created trajectories targeting the Vim and GPi and tractography, 4 targets were planned. Figure 2a depicts a reconstruction of the implanted leads while Figures 2b,c depicts personal DRTT pathways reconstructed in the patient’s native image space using FSL software, described below. The fiber tracts were reconstructed as a secondary target option if Vim anatomy was found to be too distorted for reliable lead placement. Ultimately, a decision was made to place the Vim leads with the 2 distal contacts placed deep relative to Vim borders under tractographic guidance to partially cover DRTT fibers. All leads were attached to extensions, marked appropriately, and externalized for extra-operative testing.

### Fiber-Tract Reconstruction

A T1 volumetric image (0.5 × 0.5 × 0.5 mm resolution) and diffusion weighted imaging (*b* = 1000, 33 vector directions, 2 × 2 × 2 mm resolution) were acquired preoperatively for the patient. Volumetric CT was acquired postoperatively for electrode localization. Diffusion imaging distortion correction was accomplished with TOPUP (Andersson et al., 2003). Additionally, eddy correct was used to reduce artifact (Andersson and Sotiropoulos, 2016). FSL’s bedpost preprocessing was run, and DTI analysis was performed using the pre-processed data (Behrens et al., 2003). The DRTT was reconstructed by mapping tracts from the contralateral SCP, through the thalamus, to the premotor area using FSL’s Probtrack. The resultant streamline voxel map was thresholded at 5% of the total streamlines connecting the SCP to the pre-motor area. Tracts were linearly transformed to patient specific T1 space using a 6 degree-of-freedom (DOF) transformation with FSL’s FLIRT algorithm (Jenkinson et al., 2002). The patient’s postoperative CT was also transformed to patient specific T1 space using a 6 DOF transform.

### Interrogation of Externalized Deep Brain Stimulation Leads

Both Vim and GPi targets were found to reduce tremor severity on bedside extra-operative testing. Spiral drawing and postural tremor changes were used to monitor stimulation effects. For the right-hand tremor, left Vim stimulation using bipolar configurations of all contacts demonstrated significant tremor benefits (Figure 1B). There was mild ataxia at amplitudes above 4 mA. Furthermore, there was improvement of postural tremor with left GPi stimulation at low amplitudes without capsular side effects. Similar effects were observed on the left hand during right Vim stimulation using the three most ventral contacts. However, in contrast with the left GPi, right GPi stimulation did not demonstrate added benefit to the postural left-hand tremor. There were no immediate gait effects or side effects during right GPi stimulation. No paresthesias were exhibited during programming of both Vim targets. The patient tolerated interrogation of externalized leads without complications. Given consistent benefits observed with bilateral Vim stimulation, lack of side effects to guide electrode repositioning, and benefits of postural components of the right-hand tremor, electrode locations were maintained for internalization of all DBS leads 3 days following the initial surgery. Each pair of leads (left and right) was connected to two separate IPGs (Boston Scientific Gevia) placed in the subclavicular region bilaterally.

### Post-operative Follow-Up

The patient tolerated both procedures well without complication. Electrode screening was performed over monthly post-operative sessions to isolate the effects of each target. Vim stimulation was found to significantly reduce postural and kinetic tremor of both hands. Adverse effects included significant ataxia at higher stimulation amplitudes. The addition of GPi was found to stabilize wing-beating tremor but no consistent effects were observed on kinetic tremor or gait ataxia. Follow-up at 3 and 6 months revealed further stabilization of bilateral arm tremors with higher stimulation of both Vim and GPi. The patient was able to bring food to his mouth from a bowl and use his phone whereas prior to surgery this was extremely difficult to impossible. At interim follow-up programming sessions, the patient reported ataxia with moderate voltages (2.25–2.5 mA). Vim voltage was lowered to reduce ataxia, though some degree of imbalance was still observed at minimum levels required for tremor control. In order to avoid falls but still provide tremor benefits during manual tasks such as eating, he was recommended to keep the stimulator off during prolonged ambulation. Final electrode configurations were as follows (contacts labeled 1–4 with 1 being the most ventral): Left Vim C + 2-, 2 mA, 70 μs, 150 Hz; Right Vim C + 1-, 1.5 mA 70 μs, 150 Hz; Left GPi C + 1-, 1 mA, 60 μs, 150 Hz; Right GPi C + 2-, 1.5 mA, 60 μs, 140 Hz. Pre- and post-surgery examinations were documented by the same movement disorder specialist (N.VA). Motor assessments were made using the performance subscale of The Essential Tremor Rating Assessment Scale (TETRAS) (Ondo et al., 2018), including head, right upper extremity, left upper extremity and Archimedes Spirals (scored 0–4, with 0 being least severe). These results are summarized in Table 1 and indicate a 67% improvement in head and upper extremity tremor. Figure 1C shows 6-month post-operative spiral drawing test with stimulation turned off, while Figure 1D shows spiral drawing at 6-month on stimulation.

**TABLE 1:** Performance subscale of The Essential Tremor Rating Assessment Scale (TETRAS).

**Item**	**Pre-DBS**	**6-mo Post-DBS**
Head	2	0
LUE forward outstretched	3	1
RUE forward outstretched	3	1
LUE wing beating	4	0
RUE wing beating	4	0
LUE kinetic	3.5	2
RUE kinetic	3.5	2
Archimedes Spirals L	4	2
Archimedes Spirals R	4	2
Composite sub-score	31	10

*Sub-score graded by a movement disorder neurologist on preoperative and 6-month postoperative videos. The documented composite performance improvement was found to be 67% in head and upper extremity tremor.*

## Discussion

Movement disorders are common sequelae of trauma to the brain including in cases of vascular insult and diffuse axonal injury. The effects can be widespread depending on the mechanism of injury and can involve pathways linking cerebellum, brainstem, basal ganglia, and motor cortex (Ramirez-Zamora and Okun, 2016).

Cerebellar outflow tremors are characterized by irregular oscillatory movements of the extremities with a frequency less than 5 Hz (Deuschl et al., 1998). They are typically characterized by intention tremor often with an accompanying postural tremor and lacking a resting tremor component (Deuschl et al., 1998). They normally manifest in the acute lesional period following trauma to the brain (Ghika-Schmid et al., 1997). Typical intention and postural tremor are presumably generated within the dentato-rubro-thalamo-cortical or the dentato-rubro-olivary circuits (Choi, 2016). The pathophysiology of cerebellar tremor is not yet thoroughly understood, but it is believed to be related to dysfunction of the cerebellar efferent pathways (Javalkar et al., 2014; Choi, 2016). The denomination of Holmes’ tremor has been ascribed to an unusual combination tremor syndrome involving the proximal and distal parts of the upper extremities with rest tremor that is more severe on postural maintenance and most severe at kinetic intention (Puschmann and Wszolek, 2011; Kipfer and Frigerio, 2013). There can be accompanying features of dystonia, dysmetria, and/or dysdiadochokinesia (Puschmann and Wszolek, 2011). This condition is often caused by stroke or trauma in the brainstem and usually presents weeks to years following a brain lesion (Raina et al., 2016). The kinetic/postural tremor has been attributed to cerebello-thalamic pathway damage while the resting tremor has been linked to nigrostriatal pathway damage (Puschmann and Wszolek, 2011; Javalkar et al., 2014).

While our patient presented with characteristics of both cerebellar outflow (intention/postural tremor of the extremities without resting component) and Holmes’ (delayed onset, dystonia) tremor, his trauma clearly resulted in dysfunction of diffuse pathways linked to cerebellum and basal ganglia and subsequently a complex movement disorder semiology.

Proper management of post-traumatic tremor is limited by the lack of effectiveness of pharmacological approaches, leading to a growing interest in surgical techniques (Mendonça et al., 2018; Wang et al., 2020). Several systematic reviews have been published on a small pool of patients in the literature – fewer than 100 to date – and have found that deep brain stimulation can be an effective therapeutic modality and can provide greater postural tremor suppression compared with medical management (Rojas-Medina et al., 2016; Artusi et al., 2018; Wang et al., 2020).

More commonly used targets for stimulation include Vim, GPi, VOP (Foote and Okun, 2005; Espinoza Martinez et al., 2015; Mendonça et al., 2018). Patients who develop tremor following CNS stroke [“post-stroke tremor” (PST)] have been found to have better prognoses than those with history of CNS trauma (PTT). In PST, Mendonça et al. (2018) found median tremor improvement of 80% for thalamic and 77.5% for GPi targets in a pooled cohort of 21 patients, while in PTT median improvement was found to be 66.7 and 54.4%, respectively, in 23 pooled patients. While reported outcomes may be biased by heterogeneity of surgical procedure and tremor evaluation, it is also likely that trauma to the brain results in more diffuse damage including white matter bundles responsible for coordinated movement.

Less commonly, the dentato-rubro-thalamic tract (DRTT) has been used as a target for stimulation (Coenen et al., 2011). Containing fibers that terminate in the ventral oralis and intermedius nuclei of the thalamus and that subsequently project onto motor cortex, the DRTT is critically involved in coordinating somato-motor function (Coenen et al., 2011). This approach is useful as the DRTT is a common tremor-reducing pathway and an effective target when conventional target anatomy is distorted or destroyed by prior insult (Coenen et al., 2011, 2020; Boccard et al., 2016). More commonly the DRTT has been targeted for successful treatment of more prevalent tremor syndromes including essential, Parkinson’s, and multiple sclerosis tremors (Fenoy and Schiess, 2017; Coenen et al., 2020; Kübler et al., 2021).

To localize the DRTT, DTI tractography is presently the most effective way to reliably guide navigation (Boccard et al., 2016). Table 2 includes articles describing the use of DRTT targeting for the treatment of rare, therapy-refractory acquired tremors such as PTT with available outcomes (Kudo et al., 2001; Plaha et al., 2008; Coenen et al., 2011; Morishita et al., 2019; Bargiotas et al., 2021). Still, lack of response to stimulation in canonical targets can happen in patients who have experienced diffuse anatomical damage (Boccard et al., 2016; Coenen et al., 2020). These patients can benefit from tailored surgical approaches focused on maximally treating symptoms. To accomplish this, explanted programming immediately following implantation is one method to conveniently address patient needs while mitigating the risk for stimulation response failure and repeat surgery (Kashanian et al., 2021). Lead externalization can also be beneficial when lead testing may not be reliable in the operating room or when more time is needed to isolate treatment response in complex cases such as this one. Additionally, this approach may be considered more generally in patients who cannot undergo awake DBS surgery but would benefit from lead placement optimization in an extra-operative environment. Primary concerns with the lead externalization approach center around a potential risk for extra-operative infection. This risk is mitigated with careful handling of leads cables and regular sterilization of the puncture sites during the course of trial period, which is typically no longer than a few days. A recent meta-analysis found that the rate of infection in patients with electrode externalization is comparable to that reported in the literature for DBS implantation without a trial period (Kashanian et al., 2021). While not standard of practice, this method is gaining popularity among movement disorder specialists and may be utilized more frequently as newer indications for DBS develop.

**TABLE 2:** Literature review of patients with rare, acquired tremor syndromes who underwent DBS of the DRT/DRTT.

**Authors**	***n***	**Diagnoses**	**mean FU (mo.)**	**DBS target**	**DRT/DRTT targeting method**	**Outcome data**
Bargiotas et al., 2021	4	HT (post-stroke)	48	Unilateral Vim (2) or DRTT (2)	direct	Average FTMTRS reduction at last FU*: Vim 29.25%; DRTT 15.30%
Morishita et al., 2019	7	ET (3), PD (2), HT [post-head trauma (1), post-stroke (1)]	11.1 ± 6.4	Bilateral thalamic (red nucleus & Vim) intended to engage the DRT	direct	Reductions in tremor rating scale scores for ET, PD, and HT patients were 42.63, 71.43, and 56.09%, respectively
Coenen et al., 2011	1	Myoclonic head tremor	3	Bilateral DRT	direct	ETRS score improved by 18% postoperatively; >90% control of head tremor at 3 months
Plaha et al., 2008	18	PD (5), HT (1), cerebellar tremor (1), ET (6), MS tremor (4), dystonic tremor (1)	12	Bilateral cZI (intended to engage white matter tracts surrounding the subthalamic region)	indirect	Tremor improvement for all diagnoses: PD resting 94.8%, postural 88.2%; ET 75.9%; HT 70.2%; cerebellar tremor 60.4%; MS tremor 57.2%; dystonic tremor improved both tremor and dystonia
Kudo et al., 2001	1	HT (post-stroke)	N/A	Bilateral Vim (engaging the DRTT)	indirect	almost complete abolition of tremor in both the head and arm, though some ataxia of arm still present

*Few case reports and series describing intentional targeting of dentato-rubro-thalamic circuit implicated white matter fiber tracts. Multiple targeting and tremor evaluation strategies are reported.*

*3 of 4 patients saw sustained improvement for 3 years, followed by deterioration of ADL performance to worse than baseline by 4 years; 1 of 4 patients saw sustained improvement for 9 years. FU – follow-up; HT – Holmes’ tremor; ET – essential tremor; PD – Parkinson’s disease; MS – multiple sclerosis; DRT/DRTT – dentato-rubro-thalamic tract; cZI – caudal zona incerta; FTMTRS – Fahn-Tolosa-Marin Tremor Rating Scale; ETRS – Essential Tremor Rating Scale.

## Conclusion

We present the case of a 42-year-old man with years of severe, disabling post-traumatic tremor who underwent bilateral, dual-target DBS with subsequent improvement of his complex motor symptoms as early as 3 months postoperatively and sustained progressive improvement at 6 months. A patient-tailored operative approach including target selection, extra-operative lead interrogation, and phased post-operative electrode survey was shown to be effective in optimizing clinical outcome.

## Data Availability Statement

The original contributions presented in the study are included in the article/supplementary material, further inquiries can be directed to the corresponding author.

## Ethics Statement

The studies involving human participants were reviewed and approved by the Baylor College of Medicine Institutional Review Board. The patients/participants provided their written informed consent to participate in this study. Written informed consent was obtained from the individual(s) for the publication of any potentially identifiable images or data included in this article.

## Author Contributions

RG, BS, NV, and SS designed the research and wrote the manuscript. RG, RN, AA, GB, and NV performed the research. BS, AK, ML, GB, and SS critically revised the manuscript. All authors contributed to the article and approved the submitted version.

## Conflict of Interest

SS is a consultant for Boston Scientific, Neuropace, Abbott, and Zimmer Biomet. The remaining authors declare that the research was conducted in the absence of any commercial or financial relationships that could be construed as a potential conflict of interest.

## Publisher’s Note

All claims expressed in this article are solely those of the authors and do not necessarily represent those of their affiliated organizations, or those of the publisher, the editors and the reviewers. Any product that may be evaluated in this article, or claim that may be made by its manufacturer, is not guaranteed or endorsed by the publisher.
